# The Effect of HER3 Expression on Prognosis in EGFR-Mutant Non-Small Cell Lung Cancer: A Retrospective Real-World Study

**DOI:** 10.3390/medicina62030538

**Published:** 2026-03-13

**Authors:** Canan Yıldız, Meltem Baykara, Hacer Demir, Ramazan Cosar, Sedat Yıldız, Beyza Unlu, Yaşar Culha, Duygu Ozaskin, Merve Kuday Özkan, Fariz Emrah Özkan, Çiğdem Özdemir

**Affiliations:** 1 Department of Medical Oncology, Afyonkarahisar Health Sciences University, Afyonkarahisar 03030, Turkey; meltembaykara@yahoo.com (M.B.); drhacerdemir@gmail.com (H.D.); ramazancosar@gmail.com (R.C.); drsedatyildiz06@gmail.com (S.Y.); dr-beyza@hotmail.com (B.U.); drjasar@hotmail.com (Y.C.); dygalan91@gmail.com (D.O.); mervekuday@gmail.com (M.K.Ö.); dr.emrah.oz@gmail.com (F.E.Ö.); 2 Department of Medical Pathology, Afyonkarahisar Health Sciences University, Afyonkarahisar 03030, Turkey; drcozdemir75@hotmail.com

**Keywords:** HER3, EGFR-resistance, non-small cell lung cancer

## Abstract

*Background and Objectives*: Non-small cell lung cancer (NSCLC) accounts for approximately 85% of all lung cancers and remains a leading cause of cancer-related mortality worldwide. EGFR-targeted tyrosine kinase inhibitors (TKIs) have substantially improved outcomes in EGFR-mutant NSCLC; however, primary and acquired resistance continues to limit their long-term efficacy. HER3 (receptor tyrosine-protein kinase ErbB3), a member of the ErbB receptor family, has been implicated in TKI resistance through heterodimerization with EGFR and HER2, leading to downstream PI3K/AKT pathway activation. Despite its biological plausibility as a resistance mediator, the clinical significance of HER3 expression as a prognostic and predictive biomarker in EGFR-mutant NSCLC has not been thoroughly characterized in real-world cohorts. *Materials and Methods*: This retrospective, single-center study included 52 patients diagnosed with EGFR-mutant NSCLC who received TKI therapy at Afyonkarahisar Health Sciences University between January 2011 and September 2023. HER3 protein expression was evaluated by immunohistochemistry (IHC) on formalin-fixed, paraffin-embedded tumor tissue sections using the Huabio anti-HER3 antibody (clone PD00-44, 1:2000 dilution). Staining in more than 30% of tumor cells was considered HER3-positive; membranous staining intensity was scored on a 1–3 scale. Progression-free survival (PFS1, PFS2) and overall survival (OS) were analyzed using the Kaplan–Meier method and log-rank test. Statistical significance was set at *p* < 0.05. *Results*: Of 52 patients (55.8% female; mean age 64.5 years), 59.6% received chemotherapy and 40.4% received an EGFR TKI as first-line treatment; erlotinib constituted 71.2% of targeted therapies. In the first-line TKI group, HER3-negative patients had a numerically longer median PFS1 compared with HER3-positive patients (14.0 vs. 7.1 months; *p* = 0.285); however, this difference did not reach statistical significance and should be interpreted with caution given the small sample size. In contrast, among patients receiving first-line chemotherapy, HER3 staining status did not meaningfully affect PFS1 (4.1 vs. 2.5 months; *p* = 0.063). In second-line treatment, HER3-positive patients who received TKI after prior chemotherapy demonstrated a PFS2 comparable to or slightly exceeding that of HER3-negative patients (21.8 vs. 19.8 months; *p* = 0.49), suggesting that the sequencing of chemotherapy before TKI may attenuate the adverse effect of HER3 positivity. Median OS was 15.1 months in HER3-negative patients and 12.7 months in HER3-positive patients (*p* = 0.824); this numerical difference of approximately 3 months did not reach statistical significance and should therefore be interpreted cautiously. Among patients receiving TKI in the first line, HER3-positive patients had a shorter median OS than HER3-negative patients (9.6 vs. 14.2 months), whereas those receiving TKI in the second line showed a trend toward longer OS in HER3-positive patients (20.5 vs. 17.2 months). *Conclusions*: HER3 expression was associated with reduced first-line TKI efficacy in EGFR-mutant NSCLC, suggesting a possible role for HER3 in primary TKI resistance; however, these findings are exploratory and did not reach statistical significance. The observation that HER3-positive patients who received chemotherapy before TKI demonstrated outcomes comparable to HER3-negative patients raises the hypothesis that treatment sequencing may potentially influence the impact of HER3 positivity, though this requires prospective validation before any clinical conclusions can be drawn. These results suggest that HER3 expression may warrant further investigation as a candidate biomarker for treatment sequencing decisions and as a potential therapeutic target in EGFR-mutant NSCLC. Prospective studies evaluating chemotherapy–TKI sequencing and HER3-directed agents such as patritumab deruxtecan (HER3-DXd) in HER3-positive patients are needed to confirm these preliminary observations.

## 1. Introduction

The incidence and mortality of lung cancer are increasing worldwide. Lung cancer is the most common cancer and the leading cause of cancer-related death [1]. According to the most recent GLOBOCAN 2022 data, approximately 2.48 million new lung cancer cases (12.4% of all cancer cases) and 1.82 million deaths (18.7% of all cancer-related deaths) were recorded globally, making lung cancer the most frequently diagnosed cancer and the leading cause of cancer-related death worldwide [2]. Lung cancer is broadly classified into two main subtypes: small cell lung carcinoma (SCLC) and non-small cell lung carcinoma (NSCLC). NSCLC accounts for approximately 85% of all lung cancer cases, while SCLC accounts for 15% [3,4]. NSCLC is a heterogeneous tumor class associated with poor prognosis. Despite multiple available therapeutic approaches, survival outcomes remain limited [5]. Clinical outcomes are directly linked to the cancer stage at diagnosis. Nonetheless, the advent of molecularly targeted therapies has improved the prognosis of patients with advanced NSCLC and has substantially extended progression-free survival (PFS) compared with chemotherapy [6]. Targeted therapies approved by the U.S. Food and Drug Administration (FDA) and/or the European Medicines Agency (EMA) for advanced-stage NSCLC (stage IIIB–IV) comprise tyrosine kinase inhibitors (TKIs) directed against specific targets including epidermal growth factor receptor (EGFR), anaplastic lymphoma receptor tyrosine kinase (ALK), ROS proto-oncogene receptor tyrosine kinase (ROS1), RET proto-oncogene (RET), MET proto-oncogene receptor tyrosine kinase (MET), neurotrophic receptor tyrosine kinases 1–3 (NTRK1–3), Erb-B2 receptor tyrosine kinase 2 (ERBB2/HER2), KRAS proto-oncogene GTPase (KRAS), and B-Raf proto-oncogene serine/threonine kinase (BRAF) [7,8].

Approximately 10–15% of NSCLC patients in the United States and Europe, and 30–40% of those in Asia, harbor EGFR mutations [9]. EGFR, HER2, HER3 (receptor tyrosine-protein kinase ERBB3), and HER4 are members of the ErbB receptor family [10,11,12]. These receptors form homodimers or heterodimers and activate downstream signaling cascades that induce cell growth, differentiation, and carcinogenesis [13]. EGF, transforming growth factor, amphiregulin, heparin-binding EGF-like growth factor, betacellulin, and epigen serve as EGFR ligands; epiregulin is a ligand for both EGFR and HER4; heregulin (neuregulin 1 and neuregulin 2) is a ligand for HER3 and HER4; and HER2 has no known ligand [14]. These receptors play important roles in cell survival, proliferation, angiogenesis, and metastasis in many cancer types [15]. The most common activating EGFR mutations are exon 19 deletions and the exon 21 point mutation (L858R). In EGFR-mutant lung cancers, mutant EGFR can interact with HER2 or HER3 and activate anti-apoptotic signaling pathways [16]. EGFR-targeted TKIs yield high response rates [17] and can provide long-term disease control [18].

The mechanisms associated with EGFR TKI resistance are diverse and include EGFR, MET, PIK3CA, and BRAF genomic alterations; commonly reported alterations associated with osimertinib resistance are the EGFR C797S mutation and amplifications of HER2 or MET [19]. EGFR TKI resistance arises through secondary mutations, bypass pathways (HER2 amplification, MET, PI3K/AKT, and MAPK activation), epithelial–mesenchymal transition (EMT), HER3 activation, and growth factors (EGF, VEGF, PDGF, FGF). The dimerization of HER3—particularly with HER2 and MET—generates potent proliferative signals and occupies a central role in resistance mechanisms. Consequently, HER3 is of great importance as both a resistance biomarker and a therapeutic target. Nevertheless, the underlying causes of a considerable proportion of EGFR TKI resistance remain undefined.

Receptor tyrosine-protein kinase ERBB3 (HER3) is expressed in various malignant solid tumors and has been detected in 83% of primary NSCLC tumors [20]. Some studies report this rate to be 42% [21]. This considerable variability in reported HER3 expression rates across studies is likely attributable to differences in patient populations, the antibodies and detection platforms used for immunohistochemical staining, and the positivity thresholds applied during scoring, which limits direct comparability between studies [20,21,22]. HER3 overexpression has been associated with the development of metastasis and shortened disease-free survival in NSCLC patients [22]. EGFR-mutant NSCLC exhibits higher HER3 expression compared with EGFR wild-type tumors [23]. Although increases in HER3 expression have been observed in cell lines that develop acquired resistance to EGFR TKIs in vitro, clear evidence that genomic alterations in HER3 confer resistance to EGFR TKIs in EGFR-mutant NSCLC is lacking [24]. Several studies have demonstrated that HER3 phosphorylation is observed in EGFR-mutant tumors with MET amplification, indicating that MET activates the PI3K/AKT pathway via HER3 phosphorylation [25]. Mutations arising in HER3 may affect its dimerization affinity with both HER2 and EGFR, thereby triggering diversification in signaling pathways [26].

Despite the growing body of evidence implicating HER3 in EGFR TKI resistance and tumor progression, its clinical significance as a prognostic and predictive biomarker in EGFR-mutant NSCLC has not been comprehensively evaluated in real-world patient cohorts [27,28,29]. Specifically, the influence of HER3 expression on treatment sequencing outcomes and its interaction with chemotherapy in this patient population remain poorly defined.

In light of all this information, the present study was planned to clinically examine the association between HER3 expression and TKI resistance, prognosis, and survival in patients with EGFR-mutant NSCLC. The primary outcome measure was progression-free survival under first-line TKI therapy (PFS1) with second-line progression-free survival (PFS2) and overall survival (OS) assessed as secondary outcomes.

## 2. Materials and Methods

The study was planned to include all patients diagnosed with non-small cell lung cancer who harbored EGFR mutations and were treated with a tyrosine kinase inhibitor, as recorded in the file archives of the Department of Medical Oncology, Faculty of Medicine, Afyonkarahisar Health Sciences University, between 1 January 2011, and 31 September 2023. Data required for evaluation were obtained from the hospital information system and the medical oncology clinic archives. A total of 80 patients who had received anti-EGFR therapy were initially identified. Demographic characteristics, pathological subtypes, radiological findings and staging information, treatments administered, treatment responses and durations, follow-up periods, and survival data were recorded. Some patients who had received anti-EGFR therapy were diagnosed in earlier years and did not have documented EGFR mutation testing. These patients were excluded from the study. After review of the available data, 52 patients with confirmed EGFR mutation status and available pathological material suitable for HER3 immunohistochemical evaluation were included in the final analysis. Patients lacking adequate tissue samples or complete clinical follow-up data were excluded. No formal imputation of missing data was performed; patients with incomplete data for a given variable were excluded from the corresponding analysis, and the available case numbers for each analysis are reported accordingly. The pathology blocks of these patients were examined. Data were entered into the SPSS software program (version 27); descriptive statistics were performed, and the Log-Rank test and Kaplan–Meier curves were used for survival analyses. Overall survival (OS) was defined as the time from the date of diagnosis to the date of death. Progression-free survival (PFS1, PFS2, PFS3) was calculated as the time from the treatment start date to the date of progression. Statistical significance was set at *p* < 0.05.

### Immunohistochemical Method and Evaluation

When selecting sections for immunohistochemical analysis, areas with the greatest number of tumor cells and no necrosis were preferred. Sections 4 microns thick were cut from formalin-fixed, paraffin-embedded tissue processed with 10% formaldehyde fixation and mounted onto poly-L-lysine coated slides. The HER-3 antibody (Huabio, clone PD00-44, Woburn, MA, USA) was used at a 1:2000 dilution. Antigen retrieval was performed using ER2 buffer with an incubation time of 20 min. Staining was performed using the streptavidin–peroxidase method with DAB substrate for visualization. The Leica Bond Max Automated Immunohistochemical Staining Device (Leica Microsystems, Berlin, Germany) was used for immunohistochemical staining. Stained slides were examined under an Olympus BX51 model microscope (Olympus Corporation, Tokio, Japan).

**Figure 1:**
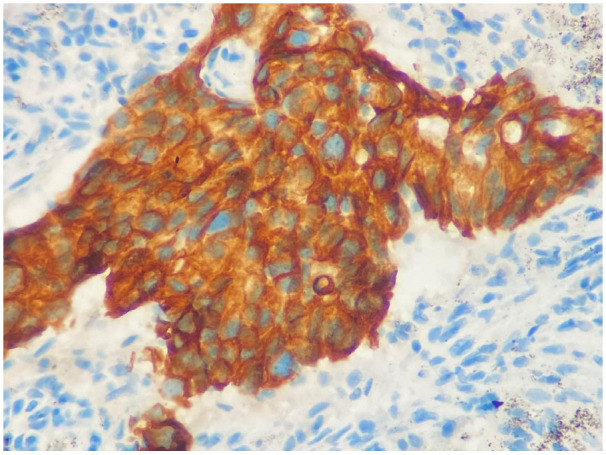
Complete membranous strong reaction, score 3 (×400).

**Figure 2:**
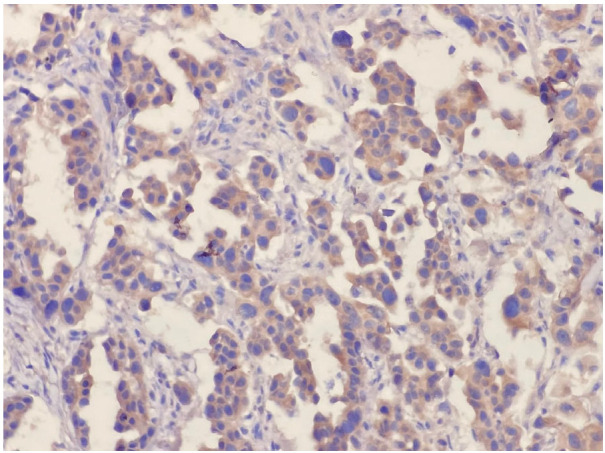
Cytoplasmic diffuse positive reaction (×200).

During HER-3 immunohistochemical staining evaluation, both positive and negative controls were taken into account. Staining in more than 30% of tumor cells was considered positive, consistent with the positivity threshold applied in previously published immunohistochemical studies evaluating HER3 expression in NSCLC [20,22]. Evaluation was based on membranous staining intensity. Weak membranous staining was scored as 1, complete but moderate membrane staining was scored as 2, and complete and strong staining was scored as 3 (Figure 1 and Figure 2). HER3 immunohistochemical evaluation was performed by two experienced pathologists with expertise in thoracic pathology at our center. The staining results were independently reviewed and scored according to predefined criteria, and any discrepancies were resolved by consensus. Formal interobserver agreement was not statistically assessed, which represents a limitation of the study.

## 3. Results

Of the 52 patients included in the study, 55.8% (*n* = 29) were female and 44.2% (*n* = 23) were male. The mean age was 64.5 years (range: 38–84). A family history of malignancy was present in 32.7% of patients (*n* = 17). Of the patients, 63.5% (*n* = 33) had never smoked, and 7.7% (*n* = 4) were former smokers. Among active smokers, 63.2% (*n* = 12) had a smoking history exceeding 30 pack-years. ECOG PS 0–1 was observed in 80.8% of patients (*n* = 42). Diagnosis was established via bronchoscopy in 78.8% of patients (*n* = 41). The most common site of metastasis was bone (53.8%; *n* = 28), followed by pleural involvement (38.5%; *n* = 20) and lymph node metastasis (36.5%; *n* = 19). In the first line, 59.6% of patients (*n* = 31) received chemotherapy and 40.4% (*n* = 21) received anti-EGFR TKI. Platinum plus pemetrexed comprised 70% (*n* = 21) of chemotherapy regimens. Erlotinib constituted 71.2% (*n* = 37) of targeted therapies (Table 1).

**Figure 3:**
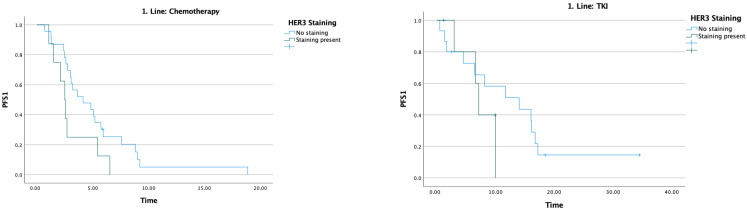
Effect of HER3 staining on first-line treatment efficacy.

The median PFS1 was 5.2 months. In patients receiving chemotherapy, PFS1 was 4.1 months in HER3-negative patients and 2.5 months in HER3-positive patients (*p* = 0.063), a difference that did not reach statistical significance. In patients receiving TKI, PFS1 was 14 months in HER3-negative patients and 7.1 months in HER3-positive patients (*p* = 0.285); this difference likewise did not reach statistical significance (Table 2) (Figure 3).

**Figure 4:**
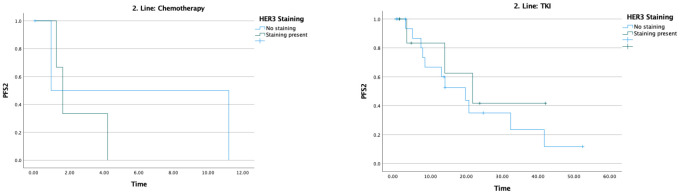
Effect of HER3 staining on second-line treatment efficacy.

The median PFS2 was 14 months. In patients receiving chemotherapy, PFS2 was 0.9 months in HER3-negative patients and 1.6 months in HER3-positive patients; these notably short values likely reflect the heavily pretreated nature of this subgroup, the small number of patients in each arm, and the real-world setting of the cohort, where performance status and available treatment options may have been more limited. In patients receiving TKI, PFS2 was 19.8 months in HER3-negative patients and 21.8 months in HER3-positive patients (Table 3) (Figure 4).

**Figure 5:**
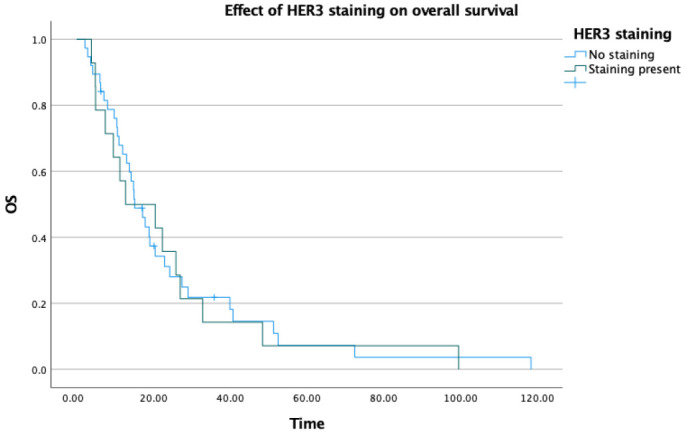
Effect of HER3 staining on overall survival.

When overall survival data were evaluated, the median OS was 15.1 months in HER3 staining-negative patients and 12.7 months in HER3 staining-positive patients. Although a numerical difference of approximately 3 months was observed, this did not reach statistical significance (*p* = 0.824) and no meaningful difference in OS between the two groups can be concluded from these data (Table 4) (Figure 5).

In the subgroup of HER3-positive patients who received chemotherapy as first-line treatment, the median OS was 20.5 months, whereas it was 9.6 months in those who received targeted therapy as first-line treatment. When the effect of second-line treatment on overall survival was evaluated, the median OS was 14.2 months in HER3-negative patients receiving chemotherapy and 17.2 months in those receiving targeted therapy. In the HER3-positive group, the median OS was 32.8 months in patients receiving chemotherapy and 20.5 months in those receiving targeted therapy.

**Figure 6:**
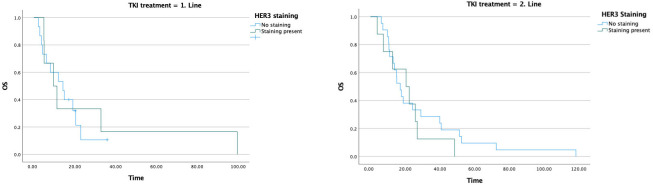
Relationship between TKI treatment line, HER3 staining status, and overall survival.

In the group that received TKI in the first line, the median OS was 14.2 months in HER3-negative patients, whereas it declined to 9.6 months in HER3-positive patients. In patients receiving TKI in the second line, the median OS was 17.2 months in HER3-negative patients and 20.5 months in HER3-positive patients (Table 5) (Figure 6).

## 4. Discussion

Human epidermal growth factor receptor 3 (HER3, or ErbB3) belongs to the human epidermal growth factor receptor (HER) family, which also includes EGFR (HER1/ErbB1), HER2 (ErbB2), and HER4 (ErbB4) [30]. Studies on HER3 biology have demonstrated that activation of HER3 signaling supports tumor progression through enhancement of metastatic potential and triggering of treatment failure in human cancers [31,32,33].

Immunohistochemical analyses of clinical specimens have shown that HER3 overexpression is associated with worse overall survival in patients with various human cancers, including colorectal cancer, gastric cancer, breast cancer, melanoma, ovarian cancer, head and neck cancer, pancreatic cancer, and cervical cancer [21]. High HER3 expression has been associated with poor prognosis and increased risk of metastasis in NSCLC [22,34,35]. Activation of HER3 signaling contributes to the drug-resistant phenotypes of HER2-positive breast cancer, castration-resistant prostate cancer, and platinum-resistant/refractory ovarian cancer [36,37,38].

Growing evidence supports HER3 as an attractive target, and HER3 inhibition is considered necessary to overcome therapeutic resistance, enhance efficacy, and improve patient survival [32,39,40,41].

It has been shown that first-generation TKIs—gefitinib and erlotinib—and the third-generation TKI Osimertinib can induce HER3 expression during the treatment of EGFR-mutant NSCLC. Up-regulation of HER3 triggers the activation of “bypass signaling pathways” through an EGFR-independent mechanism, resulting in resistance to gefitinib, erlotinib, and osimertinib [42,43,44,45,46]. The mechanisms underlying HER3 overexpression and its adaptive induction by EGFR TKIs remain poorly understood.

The present study is a real-world investigation evaluating the effects of HER3 expression on TKI resistance and PFS in patients with EGFR-mutant NSCLC.

In our study, HER3 staining status in EGFR-mutant NSCLC patients receiving chemotherapy as first-line treatment did not produce a difference in PFS as expected (4.1 months vs. 2.5 months). In contrast, among patients receiving TKI (the majority being erlotinib), the PFS was 14 months in the HER3-negative group, whereas it declined to 7.1 months in the HER3-positive group. The OPTIMAL and EURTAC trials reported median PFS of 9–11 months in the erlotinib arm [47,48]. Our finding of 14 months for TKI PFS in the HER3-negative group is consistent with these trials. In the HER3-positive staining group, PFS1 was shortened to 7.1 months, supporting TKI resistance. These results support the hypothesis that HER3 positivity may be associated with primary/early resistance to EGFR TKIs.

HER3 (ERBB3) is a receptor with weak intrinsic kinase activity that potently activates PI3K/AKT signaling through heterodimerization. Therefore, HER3 overexpression or activation may play a role in the emergence of TKI resistance by providing an alternative pro-survival pathway that bypasses EGFR blockade. Recent research has demonstrated the contribution of HER3 to TKI resistance and the potential of HER3-directed therapeutic targeting [49]. In this context, the HERTHENA-Lung01 study was a phase II clinical trial evaluating the efficacy and safety of patritumab deruxtecan (HER3-DXd, an anti-HER3 antibody–drug conjugate) in patients with EGFR-mutant advanced NSCLC who had developed resistance to treatment [50].

In the HERTHENA-Lung01 study, an objective response rate of approximately 30% and a median PFS of approximately 5.5 months were reported with HER3-DXd in EGFR-mutant NSCLC patients who had progressed after third-generation EGFR TKI, demonstrating that HER3 targeting has clinically meaningful activity in this resistant population [50]. This efficacy signal was confirmed in the randomized phase 3 HERTHENA-Lung02 trial, in which HER3-DXd achieved statistically significant PFS superiority over platinum-based chemotherapy (HR 0.77; 95% CI 0.63–0.94; *p* = 0.011), with an ORR of 35.2% [51,52]. Although median PFS values were similar between both studies, the marked divergence in 6–12-month PFS rates observed in HERTHENA-Lung02 suggests that HER3-DXd can provide long-term disease control in a specific patient subgroup. Taken together, these data confirm that the efficacy observed in HERTHENA-Lung01 is clinically meaningful and corroborated by HERTHENA-Lung02, positioning HER3-DXd as a potential alternative to platinum-based chemotherapy following EGFR TKI therapy.

When second-line treatment outcomes were evaluated, the median PFS2 was 14 months. As expected, PFS2 was markedly shortened in the chemotherapy arm regardless of HER3 staining status (0.9 months in HER3-negative patients, 1.6 months in HER3-positive patients). However, in contrast to first-line treatment, in the targeted therapy arm, PFS2 was 19.8 months in HER3-negative patients and similarly 21.8 months in HER3-positive patients. HER3 positivity is associated with resistance and is generally a poor prognostic marker. However, these patient groups had received chemotherapy as first-line treatment. In HER3-positive patients, targeted therapy administered after first-line chemotherapy may produce better outcomes than initiating targeted therapy as first-line treatment. These findings suggest a possible explanation: in HER3-positive patients who received chemotherapy as first-line treatment, prior chemotherapy may have altered the clonal tumor architecture or reduced the HER3-dependent cell population, potentially enhancing responses to subsequent targeted therapies (e.g., TKIs or other targeted agents). However, this interpretation should be considered hypothesis-generating and requires prospective validation [47,48]. No study evaluating this has been designed to date; however, the observations of our study are supportive of this notion.

Although HER3 positivity demonstrated better performance in the second-line setting, when overall survival data were evaluated, the median OS was 15.1 months in HER3-negative patients and 12.7 months in HER3-positive patients. This numerical difference of approximately 3 months did not reach statistical significance (*p* = 0.824) and should be interpreted cautiously. The finding of worse survival in HER3-positive patients is consistent with the literature [53].

Similarly, in the group receiving TKI in the first line, the median OS was 14.2 months in HER3-negative patients, whereas it declined to 9.6 months in HER3-positive patients—another finding consistent with primary TKI resistance mediated by HER3 status in the HER3-positive group. In the group receiving TKI in the second line, the median OS was 17.2 months in HER3-negative patients and 20.5 months in HER3-positive patients. In HER3-positive patients, initiating treatment with chemotherapy rather than TKI in the first line may have a favorable impact on survival, likely due to potential TKI resistance.

This study is important in highlighting the evaluation of TKI–chemotherapy combinations to overcome primary TKI resistance in HER3-positive patient groups. Phase II data support the efficacy of HER3-targeting antibody–drug conjugates (ADCs) in the post-EGFR-TKI setting; if the phase III program (HERTHENA-Lung02) confirms the superiority of this agent over platinum-based chemotherapy, HER3 positivity may become both a prognostic and a therapeutically guiding biomarker in clinical practice [53].

Our study has several limitations that must be carefully considered when interpreting the findings. First, the relatively small sample size (*n* = 52) and the retrospective, single-center design substantially reduce statistical power, particularly in subgroup analyses. As a result, several comparisons that showed clinically meaningful numerical differences—such as the 6.9-month gap in PFS1 between HER3-negative and HER3-positive patients in the first-line TKI group—did not reach statistical significance (*p* = 0.285). These findings should therefore be regarded as exploratory observations from a real-world cohort rather than definitive evidence. Second, given the limited sample size (*n* = 52) and the small number of events, a reliable multivariable Cox regression analysis could not be performed without a substantial risk of model overfitting; such an analysis would require an adequate number of events per variable to yield meaningful and stable estimates. Consequently, potential confounding factors—including ECOG performance status, specific TKI generation, and EGFR mutation subtype—cannot be excluded as contributors to the observed survival differences. Future studies with larger, prospectively collected datasets should incorporate multivariable Cox regression analyses to properly adjust for such confounders. Third, HER3 expression was evaluated solely by immunohistochemistry, without assessment of HER3 phosphorylation status, activation, or concurrent molecular alterations such as MET amplification and ERBB3 mutations, which limits a comprehensive understanding of HER3’s biological role. Fourth, the 11-year study period (2011–2023) introduced considerable treatment heterogeneity: patients received different generations of EGFR TKIs, different chemotherapy regimens, and were managed under evolving treatment guidelines, making direct comparisons across subgroups difficult to interpret. The predominant use of first-generation TKIs (erlotinib, 71.2%), with limited representation of third-generation agents such as osimertinib, further limits the generalizability of these findings to current clinical practice, where osimertinib is the standard first-line treatment for EGFR-mutant NSCLC. These limitations collectively highlight the need for prospective, multicenter studies with larger cohorts and standardized treatment protocols to validate the prognostic and predictive value of HER3 expression in this patient population.

Our study is notable for evaluating the impact of HER3 expression on clinical outcomes with real-world data in EGFR-mutant NSCLC, and for examining the effects of HER3 expression not only on overall survival but also on treatment line, treatment sequence, and progression-free survival parameters—assessments that are important for guiding clinical oncology practice. The finding that survival outcomes differed clinically meaningfully according to HER3 status in first- and second-line treatments suggests that HER3 may be not only a prognostic but also a potential predictive biomarker capable of guiding treatment strategy. Nevertheless, given the limited sample size and the absence of multivariable analysis, the current results must be interpreted as hypothesis-generating, exploratory observations derived from a real-world cohort. The statistically non-significant differences observed across several comparisons preclude definitive conclusions and underscore the need for prospective validation in larger, adequately powered studies.

## 5. Conclusions

TKI efficacy was numerically reduced in HER3-positive patient groups in the first-line setting, though this difference did not reach statistical significance. In particular, TKI administered after chemotherapy in the HER3-positive group showed numerically better performance, raising the hypothesis that treatment sequencing may potentially influence outcomes. In second-line targeted therapy, this difference disappears or appears to reverse. These preliminary, exploratory data suggest that HER3 expression may represent a potential candidate biomarker and therapeutic target warranting further prospective investigation, though no definitive conclusions regarding its predictive value can be drawn from this study alone. When overall survival data are evaluated, a numerical trend toward shorter survival times in the HER3-positive group was observed, consistent with the literature, though statistical significance was not reached.

The findings of this exploratory study suggest that HER3 expression in EGFR-mutant NSCLC may potentially be associated with an adverse effect on first-line EGFR TKI response and progression-free survival, though these observations did not reach statistical significance and should be interpreted with caution. The numerical reduction in TKI efficacy in HER3-positive patients raises the hypothesis that HER3 may play a possible role in primary or early EGFR TKI resistance. The observation that numerically better outcomes were obtained with EGFR TKI therapy administered after first-line chemotherapy in HER3-positive patients suggests that individualizing the treatment sequence according to HER3 status may be worthy of prospective investigation. The numerical trend toward shorter survival times in HER3-positive patients on overall survival analyses, though not statistically significant, is consistent with the existing literature. Taken together, these results suggest that HER3 expression may represent a candidate biomarker and potential therapeutic target in EGFR-mutant NSCLC; however, prospective, adequately powered studies are needed to validate this hypothesis before any clinical recommendations can be made.

In conclusion, the findings of this exploratory study suggest that HER3 expression may potentially play a role as a resistance biomarker and therapeutic target in EGFR-mutant NSCLC; however, given the limitations of the retrospective design and small sample size, prospective studies are needed to evaluate the clinical utility of treatment sequencing according to HER3 status and to assess HER3-targeted agents in this patient population.

## Figures and Tables

**Table 1:** General characteristics (*n*: 52, %).

Variable	*n* (%)	Variable	*n* (%)
Age	64.5 (38–84)	<65 years ≥65 years	*n*: 24 (46.2%) *n*: 28 (53.8%)
Male Female	*n*: 23 (44.2%) *n*: 29 (55.8%)	ECOG PS 0–1 2–3	*n*: 42 (80.8%) *n*: 10 (19.2%)
Smoking Never Active smoker <30 pack-years ≥30 pack-years	*n*: 33 (63.5%) *n*: 7 (13.5%) *n*: 12 (23.1%)	Diagnostic Method Bronchoscopy CT-guided biopsy Other	*n*: 41 (78.8%) *n*: 6 (11.5%) *n*: 5 (9.6%)
ECOG PS 0–1 2–3	*n*: 42 (80.8%) *n*: 10 (19.2%)	Metastasis Site Bone Pleural involvement Lymph node Brain Contralateral lung	*n*: 28 (53.8%) *n*: 20 (38.5%) *n*: 19 (36.5%) *n*: 16 (30.8%) *n*: 8 (15.4%)
1st-line Treatment Chemotherapy TKI	*n*: 31 (59.6%) *n*: 21 (40.4%)	2nd-line Treatment Chemotherapy TKI	*n*: 31 (59.6%) *n*: 21 (40.4%)
3rd-line Treatment Chemotherapy TKI	*n*: 7 (70.0%) *n*: 2 (20.0%)	4th-line Treatment Chemotherapy TKI	*n*: 7 (19.4%) *n*: 29 (80.6%)
Targeted Therapy Erlotinib Afatinib Gefitinib	*n*: 37 (71.2%) *n*: 14 (26.9%) *n*: 1 (1.9%)		

**Table 2:** Effect of HER3 staining on PFS1 in first-line treatment.

1st-Line Treatment	HER3 Staining	Median Duration (Months) (95% CI)	HR	*p*
Chemotherapy	No staining	4.133 (1.577–6.690)	1.30	0.063
	Staining present	2.500 (1.853–3.147)	0.33	
	Overall	3.200 (1.709–4.691)	0.76	
TKI	No staining	14.033 (3.486–24.580)	5.38	0.285
	Staining present	7.133 (5.988–8.278)	0.58	
	Overall	10.000 (3.954–16.046)	3.08	
Overall	Overall	5.200 (3.368–7.032)	0.93	

**Table 3:** Effect of HER3 staining on second-line treatment efficacy.

2nd-Line Treatment	HER3 Staining	Median Duration (Months) (95% CI)	HR	*p*
Chemotherapy	No staining	0.933		0.61
	Staining present	1.600 (1.013–2.187)	0.29	
	Overall	1.600 (0.813–2.387)	0.40	
TKI	No staining	19.800 (8.866–30.734)	5.57	0.49
	Staining present	21.800 (5.594–38.006)	8.26	
	Overall	19.800 (11.113–28.487)	4.43	
Overall	Overall	14.033 (9.622–18.444)	2.25	

**Table 4:** Effect of HER3 staining on overall survival.

HER3 Staining	Median OS (Months) (95% CI)	HR	*p*
No staining	15.167 (10.920–19.414)	2.16	0.824
Staining present	12.767 (0.000–29.695)	8.63	
Overall	15.167 (9.859–20.475)	2.70	

**Table 5:** Relationship between TKI treatment line, HER3 staining status, and overall survival.

TKI Treatment Line	HER3 Staining	Median OS (Months) (95% CI)	HR	*p*
1st line	No staining	14.233 (5.649–22.817)	4.38	0.803
	Staining present	9.600 (1.998–17.202)	3.87	
	Overall	12.000 (5.072–18.928)	3.53	
2nd line	No staining	17.200 (12.814–21.586)	2.23	0.521
	Staining present	20.533 (7.228–33.838)	6.78	
	Overall	17.900 (11.336–24.464)	3.34	
3rd line	No staining	27.500		
	Overall	27.500		
Overall	Overall	15.167 (10.451–19.883)		

## Data Availability

The original contributions presented in this study are included in the article/Supplementary Material. Further inquiries can be directed to the corresponding author.

## Supplementary Materials

The following supporting information can be downloaded at: https://www.mdpi.com/article/10.3390/medicina62030538/s1, Kaplan-Meier curves display OS and PFS data in SPSS format.
